# Supplementary material for: Fatty acid oxidation fuels glioblastoma radioresistance with CD47-mediated immune evasion
Source: Nat Commun. 2022 Mar 21;13:1511. doi: 10.1038/s41467-022-29137-3 (PMC8938495; doi:10.1038/s41467-022-29137-3)
Supplement: Supplementary file 1 — Supplementary Information [file 41467_2022_29137_MOESM1_ESM.pdf]

Supplemental Figures

Fatty acid oxidation fuels glioblastoma radioresistance with CD47-mediated immune evasion

Nian Jiang, Bowen Xie, Wenwu Xiao, Ming Fan, Shanxiu Xu, Yixin Duan, Yamah Hamsafar, Angela C. Evans, Jie Huang, Weibing Zhou, Xuelei Lin, Ningrong Ye, Siyi Wanggou, Wen Chen, Di Jing, Ruben C. Fragoso, Brittany N. Dugger, Paul F. Willson, Matthew A. Coleman, Shuli Xia, Xuejun Li, Lun-Quan Sun, Arta M. Monjazeb, Aijun Wang, William J. Murphy, Hsing-Jien Kung, Kit S. Lam, Hong-Wu Chen, and Jian Jian Li

Supplementary Figure 1

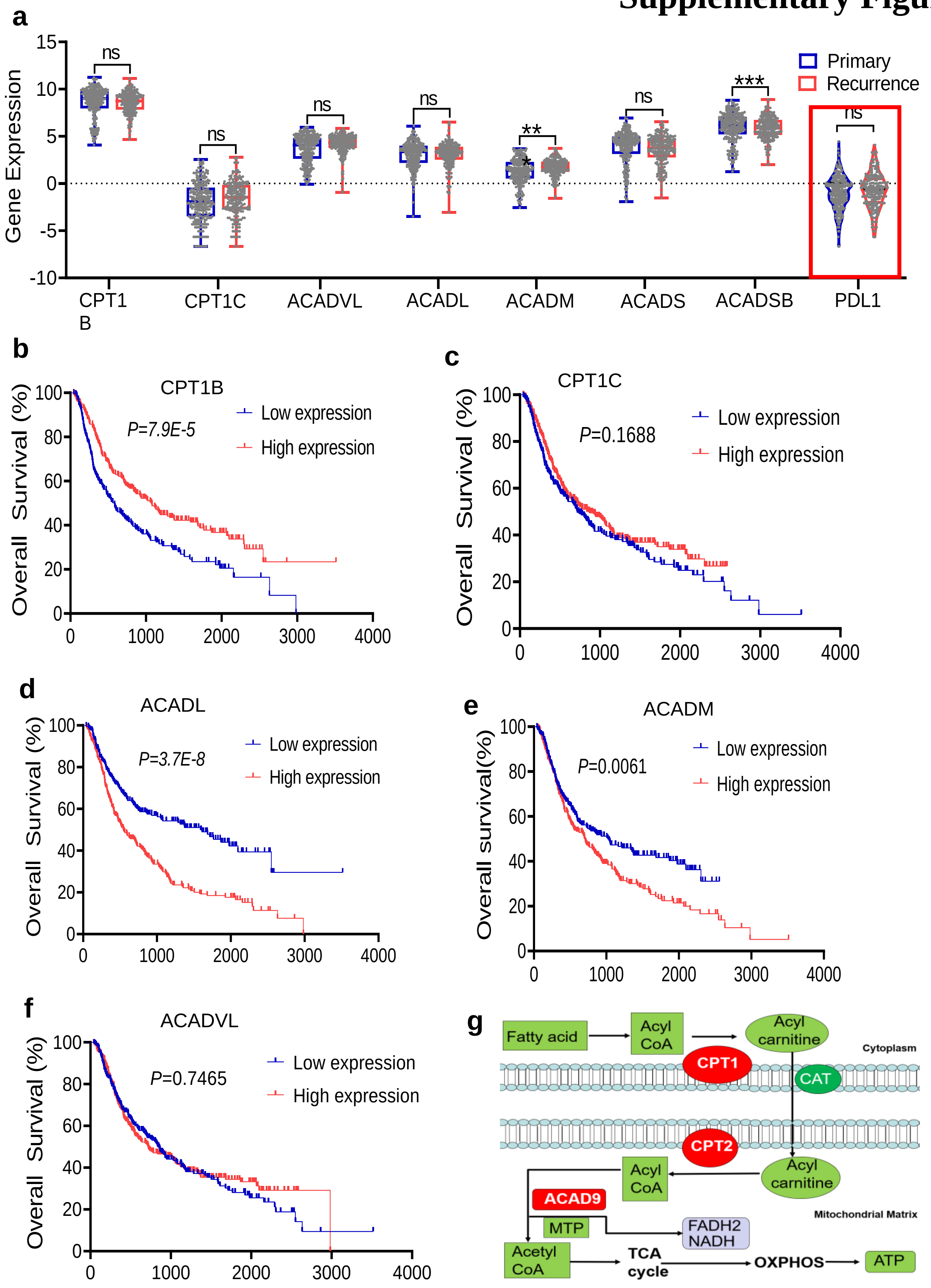

Supplementary Figure 1. Enhanced FAO enzymes in recurrent GBM with poor prognosis.

(**a**) Expression of FAO enzymes (CPT1B, *P* = 0.0723; CPT1C, *P* = 0.5205; ACADVL, *P* = 0.7480; ACADL, *P* = 0.0803; ACADM, *P* = 0.00010; ACADS, *P* = 0.8467; ACADSB, *P* = 0.0002) and PDL1 (box marked, *P* = 0.1093) in 284 primary and 220 recurrent HGG patients from CGGA database. (n= 284 in primary group; n=220 in recurrent group; ***$P < 0.001$, ns= not significant). The box represent the 25th and 75th percentile, lines show medians and error bars depict depict 1.5X IQR. Unpaired two-tailed t test was applied. Kaplan-Meier survival of 504 HGG patients categorized by high (red, n = 252) or low (blue, n = 252) with expression of CPT1B (**b**), CPT1C (**c**), ACADL (**d**), ACADM (**e**), ACADVL (**f**). Kaplan-Meier survival analysis was applied. Results represent means ± SD;; ***$P < 0.001$; ns = no significance. Results represent means ± SD. (**g**) Schematic diagram of FAO enzymes in the mitochondrial fatty acid β oxidation. Source data are provided as a Source Data file.

Supplementary Figure 2

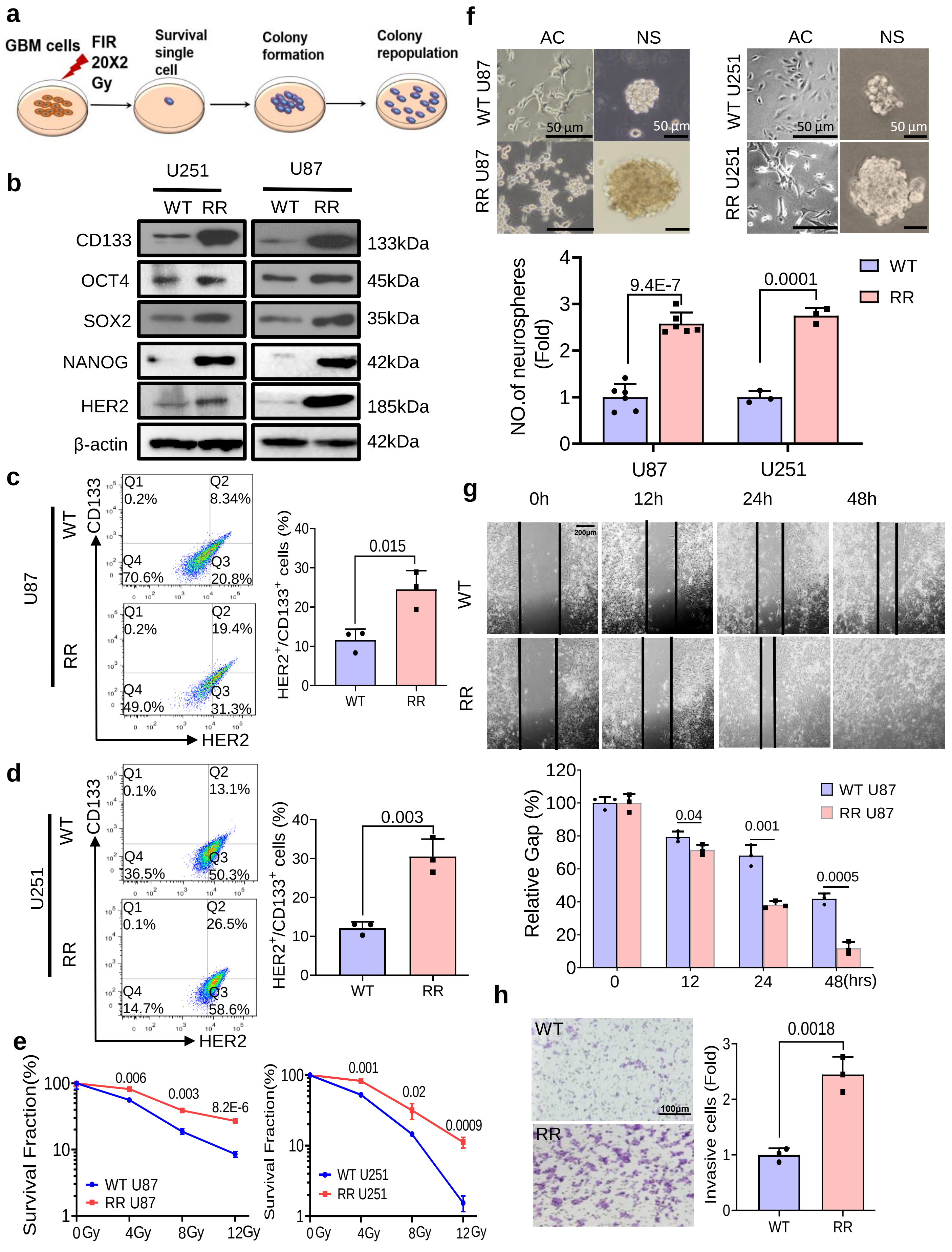

Supplementary Figure 2. Enriched HER2^{+}/CD133^{+} glioblastoma stem cells (GSCs) and enhanced aggressiveness in radioresistant (RR) GBM cells. (**a**) Schematic diagram of RR GBM cells generated by fractionated ionizing radiation (FIR; 2 Gy x 20; total dose = 40 Gy). The radioresistant colonies derived from human and mouse GBM cell lines were pooled and applied for the current experiments within 12 passages after collection. (**b**) Expression of GSC biomarkers CD133, OCT4, SOX2 and NANOG as well as HER2 detected by Western blot in RR U251 and RR U87 cells compared with parental wildtype (WT) cells (n = 3 experiments). Enhanced HER2^{+}/CD133^{+} GSCs in RR U87 (**c**) and RR U251 (**d**) cells detected by flow cytometry (n = 3). (**e**) Increased clonogenic survival of RR U87 and RR U251 cells compared with the counterpart parental GBM cells following radiation with different doses (n = 3). (**f**) Attached growth or neurosphere formation of RR and WT U87, U251 cells cultured in adherent culture (AC) medium or neurosphere culture (NS) medium (scale bar = 50 µm); lower panel shows the quantitation of neurospheres in RR and WT cells (n = 6 independent experiments in U87 cells ; n= 3 independent experiments in U251 cells). Gap filling (**g**) and transwell invasion (**h**) of RR and WT U87 cells (n = 3; scale bar = 200µm in **g**; scale bar = 100µm in **h**). Two-tailed t test was applied in **c-h.** Results represent means ± SD. Source data are provided as a Source Data file.

Supplementary Figure 3

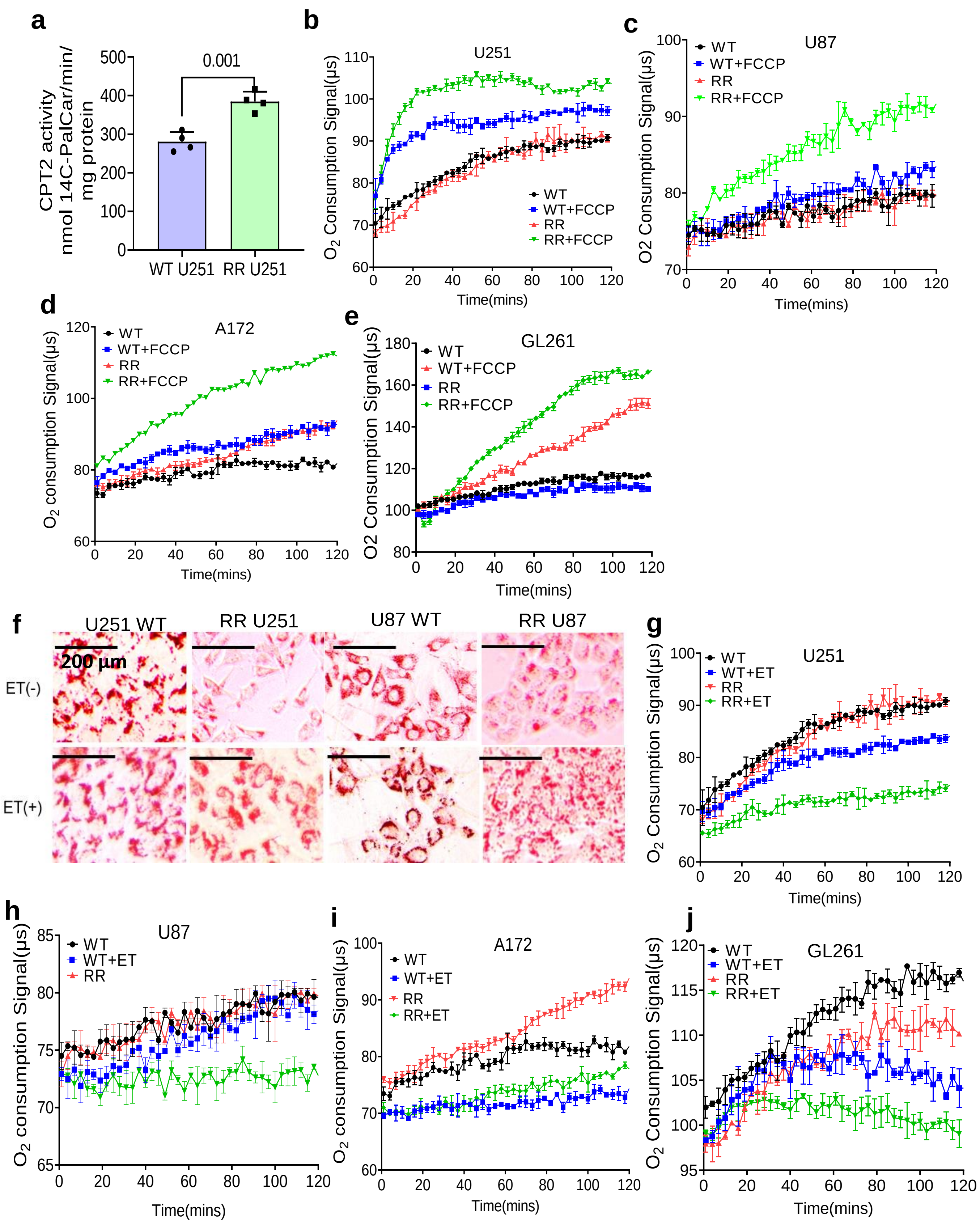

Supplementary Figure 3. Mitochondrial lipid metabolism dominates in RR GBM cells.

(**a**) CTP2 enzymatic activity in WT and RR U251 cells measured by monitoring ^{14}C- palmitoyl-carnitine (n = 4; means ± SD; Two-tailed t test; *P <0.05). Basal and maximal mitochondrial oxygen consumption rate (OCR) of WT and RR U251 (**b**), U87 (**c**), A172 (**d**), GL261 (**e**) cells with or without respiration activation by mitochondrial uncoupler FCCP (2.5 µM, 0.5 hr) (n=4 in **b**–**e;** results represent means ± SD). (**f**) Representative images of cytoplasmic lipid accumulation detected by oil red staining in U251 and U87 cells treated by CPT1A inhibitor Etomoxir (ET; 200 µM, 24 hrs), indicating a reduction of cytoplasmic lipids that can be restored by ET in RR GBM cells contrasted with no changes in the WT cells (scale bar = 200 µm) (n = 4). OCR of WT and RR U251 (**g**), U87 (**h**), A172 (**i**), GL261 (**j**) cells treated with or without CPT1 inhibitor Etomoxir (ET, 40 µM 0.5 hr) (n=4 in **g**–**j**; results represent means ± SD). Source data are provided as a Source Data file.

Supplementary Figure 4

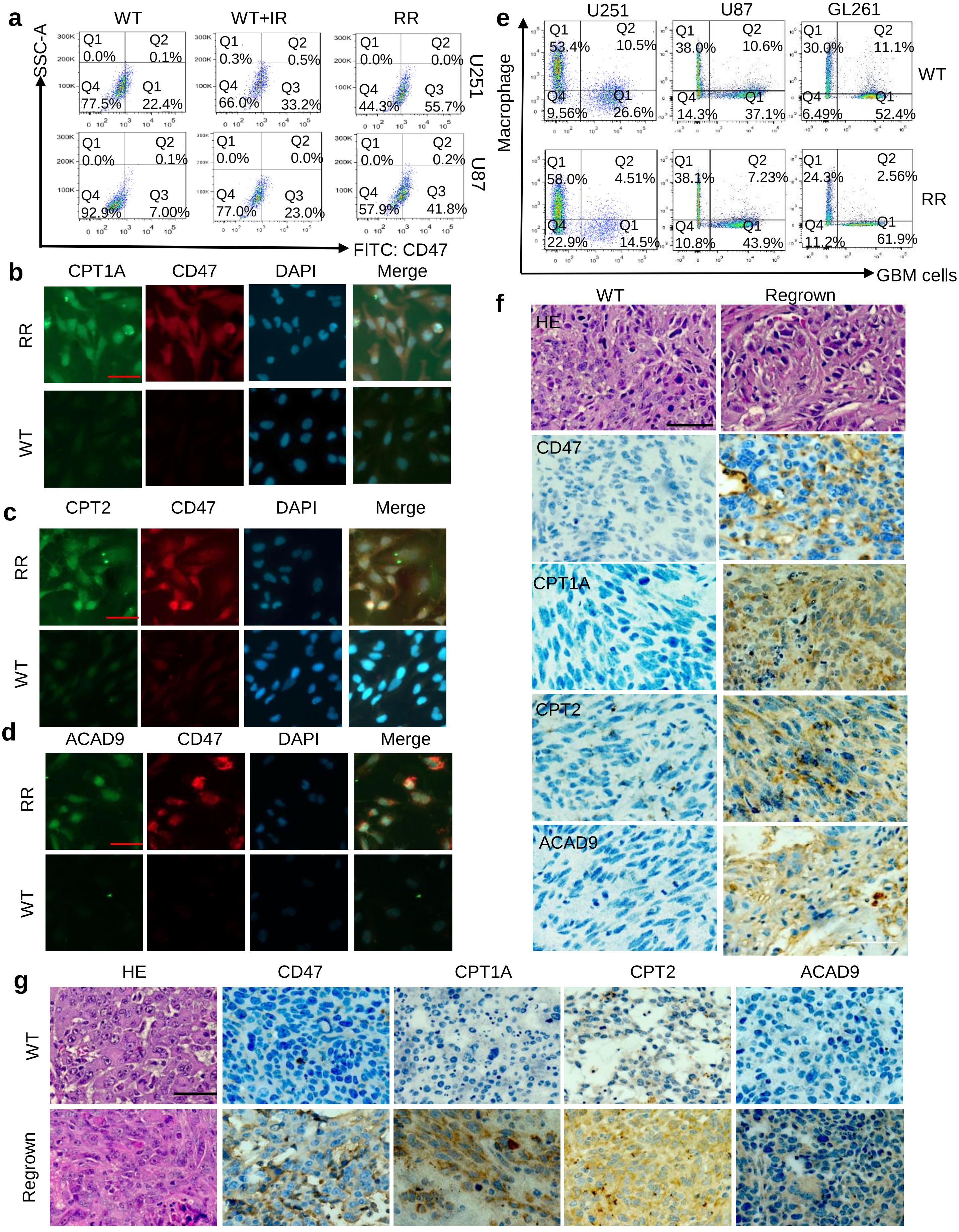

Supplementary Figure 4. Co-enhancement of CD47 and FAO enzyme expression. (**a**) flowcytometry of CD47-expressing cell population in WT, WT irradiated (5 Gy, 16 h), and RR U251 or U87 cells. Representative immune fluorescence (IF) images indicating the colocalized expression of CD47 with FAO gene CPT1A (**b**), CPT2 (**c**) ACAD9 (**d**) in RR U251 cells (n = 3, scale bar = 100 µm) . (**e**) Flowcytometry analysis of macrophage-mediated phagocytosis on WT and RR U251 and U87 cells co-cultured with activated human macrophages THP1. (**f**) Representative IHC of CD47, CPT1A, CPT2, and ACAD9 in untreated or irradiated and regrown GBM U251 tumors (scale bar = 200 µm; n = 4). (**g**) Representative IHC of CD47, CPT1A, CPT2, and ACAD9 in untreated control and the in vivo regrown mouse orthotopic GL261 tumors treated with fractionated doses of radiation (3 Gy x 3) (scale bar = 200 µm; n = 5).

Supplementary Figure 5

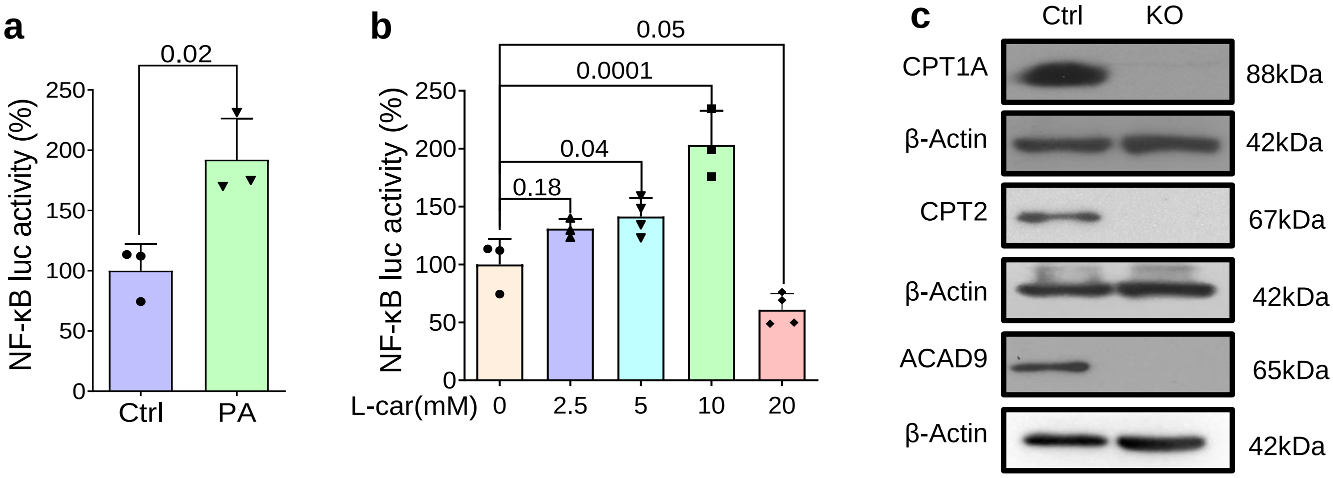

Supplementary Figure 5. FAO upregulates CD47 transcription via citrate mediated RelA acetylation. NF-κB luciferase activity in WT U251 cells treated with palmitate (PA, 25 μM, 48 hrs; **a**), or FAO enhancer L-carnitine with indicated concentrations for 48 hrs; **b**) (n = 3; Results represent means ± SD; Two-tailed t test in **a**; ANOVA one-way test in **b**). (**c**) Identification of lack of expression of CRISPR/Cas9 knocked out CPT1A, CPT2, ACAD9 in RR U251cells (n = 3). Source data are provided as a Source Data file.

Supplementary Figure 6

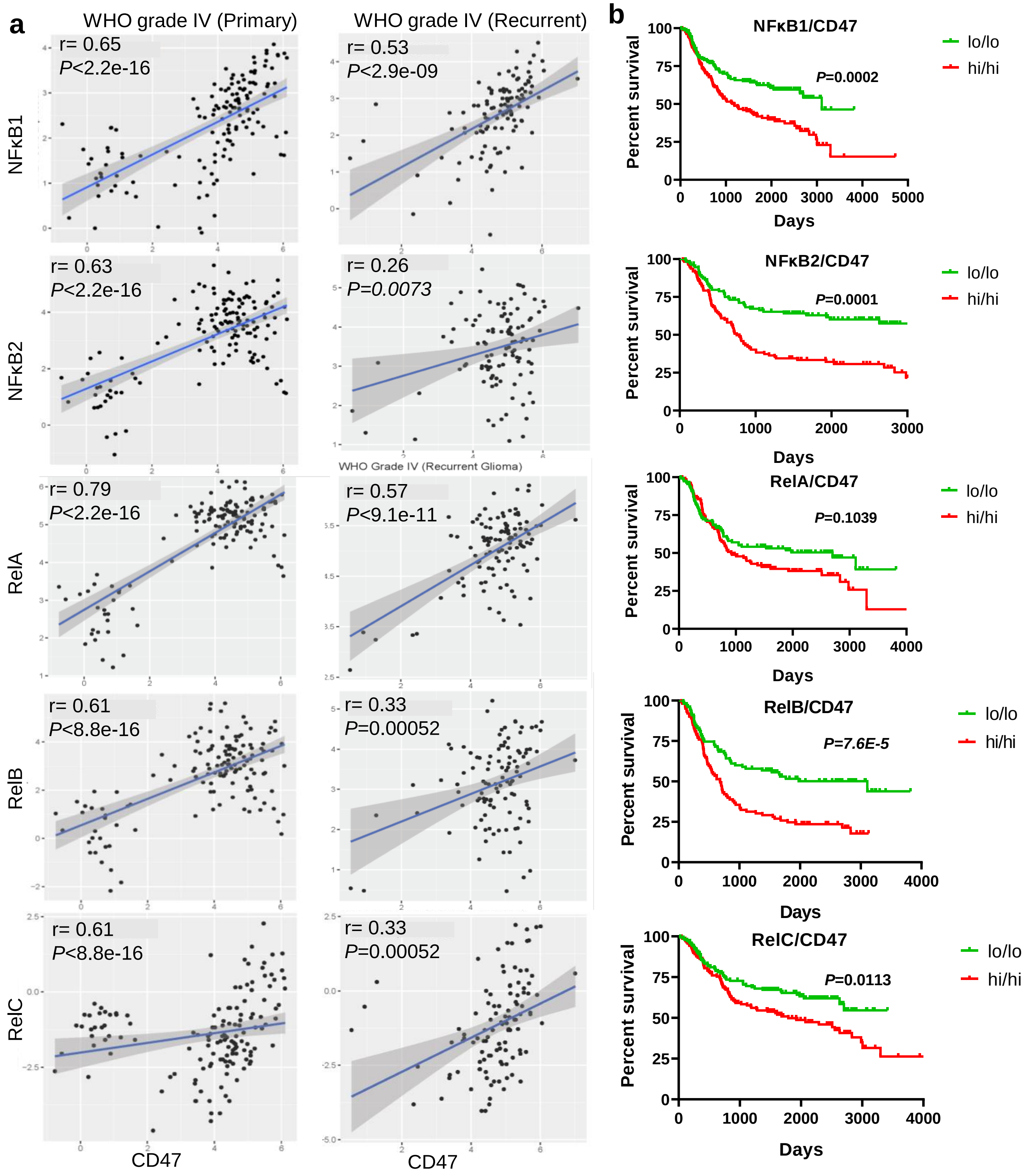

Supplementary Figure 6. Correlation between CD47 and NF-κB. (**a**) Diagrams reflecting the Pearson correlation between CD47 and the NF-κB subunits NF-κB1, NF-κB2, RelA, RelB, RelC in primary or recurrent GBM tumors from CGGA database. Pearson correlation coefficient (r) and *P*-values from Pearson correlation tests were shown. **(b)** Kaplan-Meier survival of 404 primary glioma categorized by high (red, n = 202) or low (green, n = 202) with expression of CD47, co-high expression or low expression of NF-κB subunits/CD47. Kaplan–Meier survival analysis was applied. Source data are provided as a Source Data file.

Supplementary Figure 7

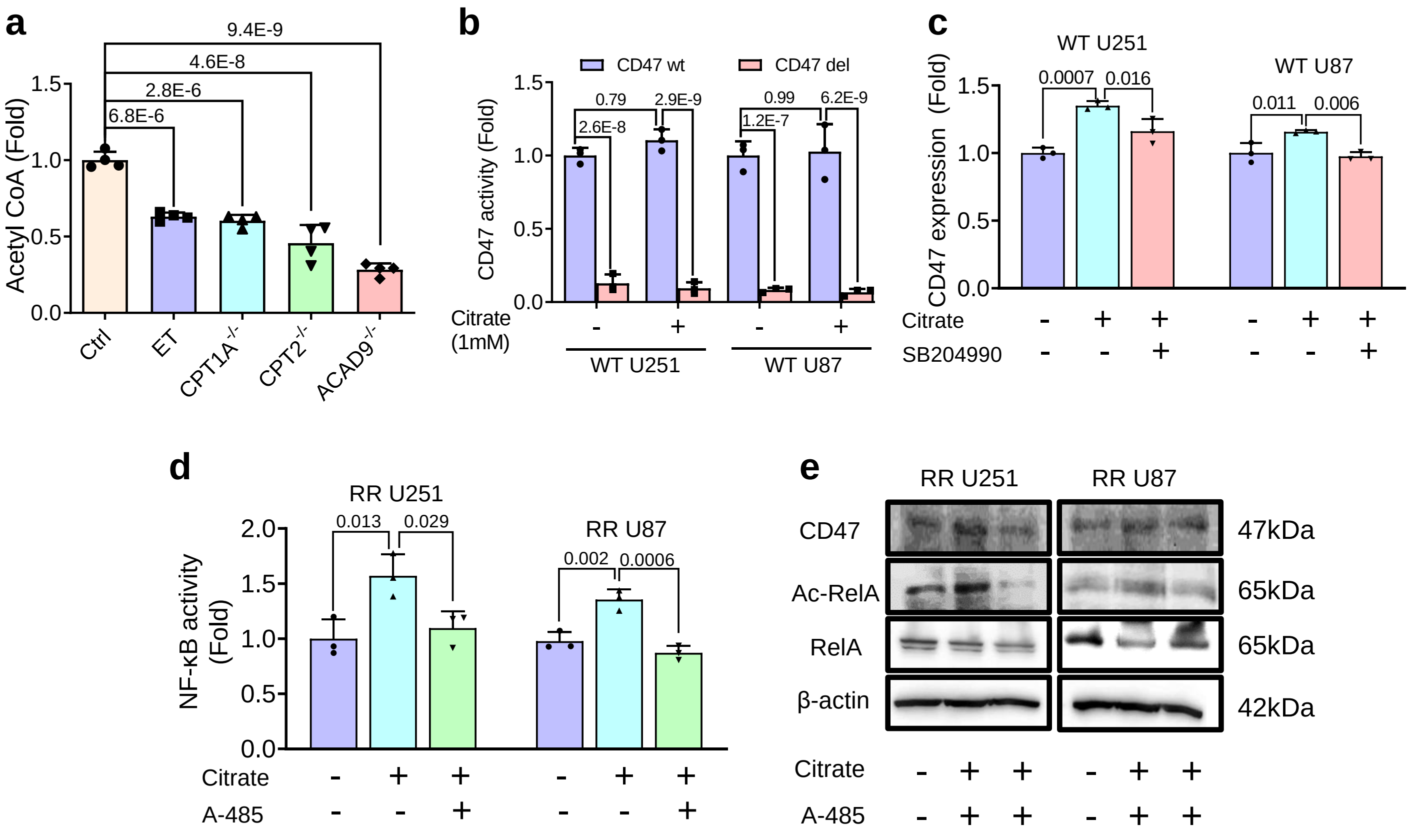

Supplementary Figure 7. FAO upregulates CD47 transcription via citrate mediated RelA acetylation. (**a**) Acetyl CoA level presented in RR U251 cells compared with ET treated (200μM, 48hrs) and CPT1A$^{-/-}$, CPT2$^{-/-}$, ACAD9$^{-/-}$ RR U251 cells (n = 4; ANOVA one-way test). (**b**) CD47 promoter-controlled luciferase activity with or without NF-κB motif deletion measured in WT U251 and WT U87 cells treated with citrate (1 mM, 6 hrs) (n = 3; ANOVA two-way test). (**c**) CD47 mRNA level in WT U251 and WT U87 cells treated with citrate (1 mM, 6 h) with or without ACLY inhibitor SB204990 (25 μM, 24 hrs) (n = 3; ANOVA one-way test) . (**d**) NF-κB luciferase activity in RR U251 and RR U87 cells treated with citrate (1 mM, 6 hrs) with or without A485 (20 μM, 24 hrs) treatment) (n = 3; ANOVA one-way test) . In **a-d**, Results represent means ± SD. (**e**)Western blot of CD47, RelA-K310 acetylation, RelA in RR U251 and RR U87 cells treated with citrate (1 mM, 6 hrs) with or without A485 (20 μM, 24 hrs) (n = 3). Source data are provided as a Source Data file.

Supplementary Figure 8

a

Human CD47 potential Acetylation sets			
**KAT**	**Site**	**Sequence**	**P-value**
CBP/p300	166	GQFGIKTLKYRSGGMDE	0.0395
CBP/p300	163	LFWGQFGIKTLKYRSGG	0.5121
CBP/p300	175	YRSGGMDEKTIALLVAG	0.884
p300	290	QLLGLVYMKFVE-----	1

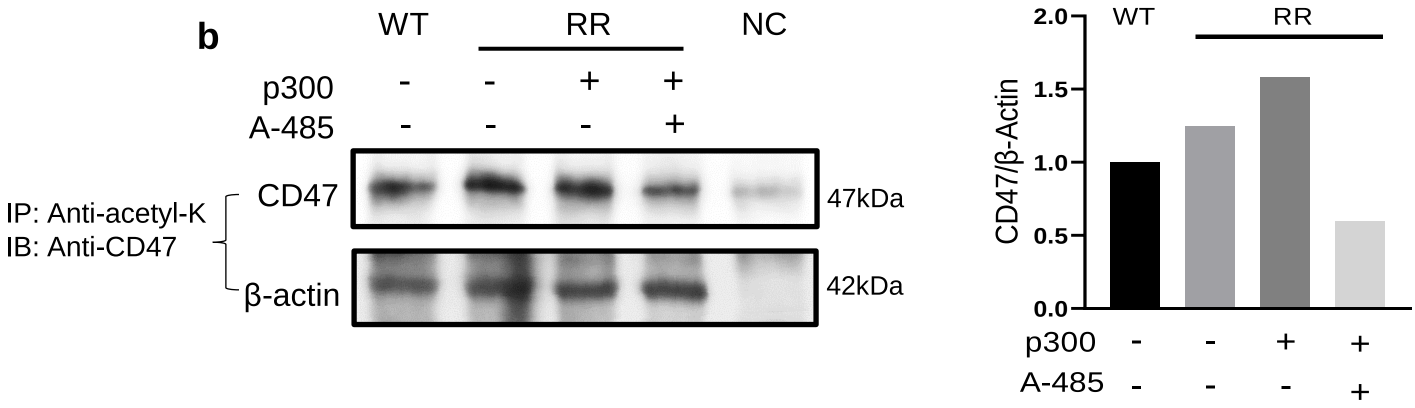

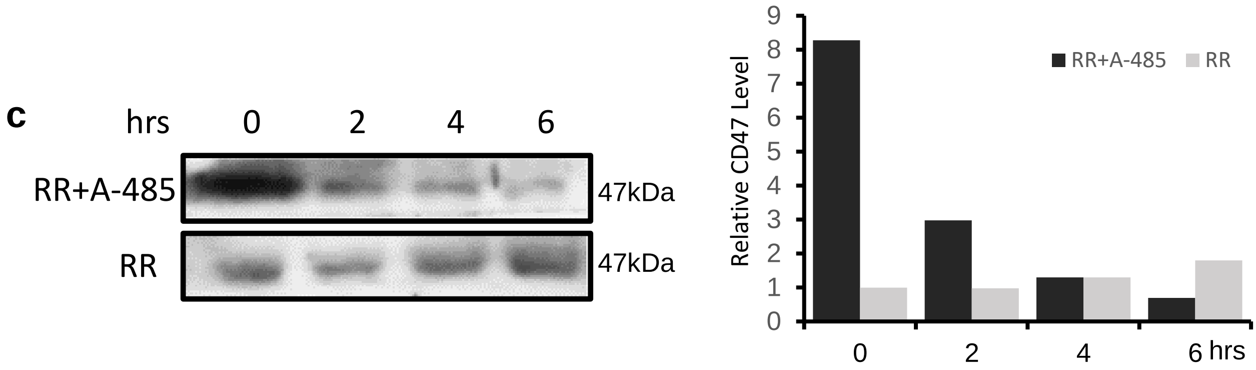

Supplementary Figure 8. (a) Predicted KAT-specific acetylation sites in human CD47 proteins. The P-value represent the chance that the given peptides were acetylated by the KAT family. (**b**) Co-immunoprecipitation assays performed with WT U251 and RR U251 cells transfected with p300 with or without A-485 (20 µM, 24 hrs) (n= 1).(**c**) Western blot of CD47 protein level in RR U251 cells with or without A-485 treatment on indicated time point after 10uM cycloheximide treatment(n= 1). Source data are provided as a Source Data file.

Supplementary Figure 9

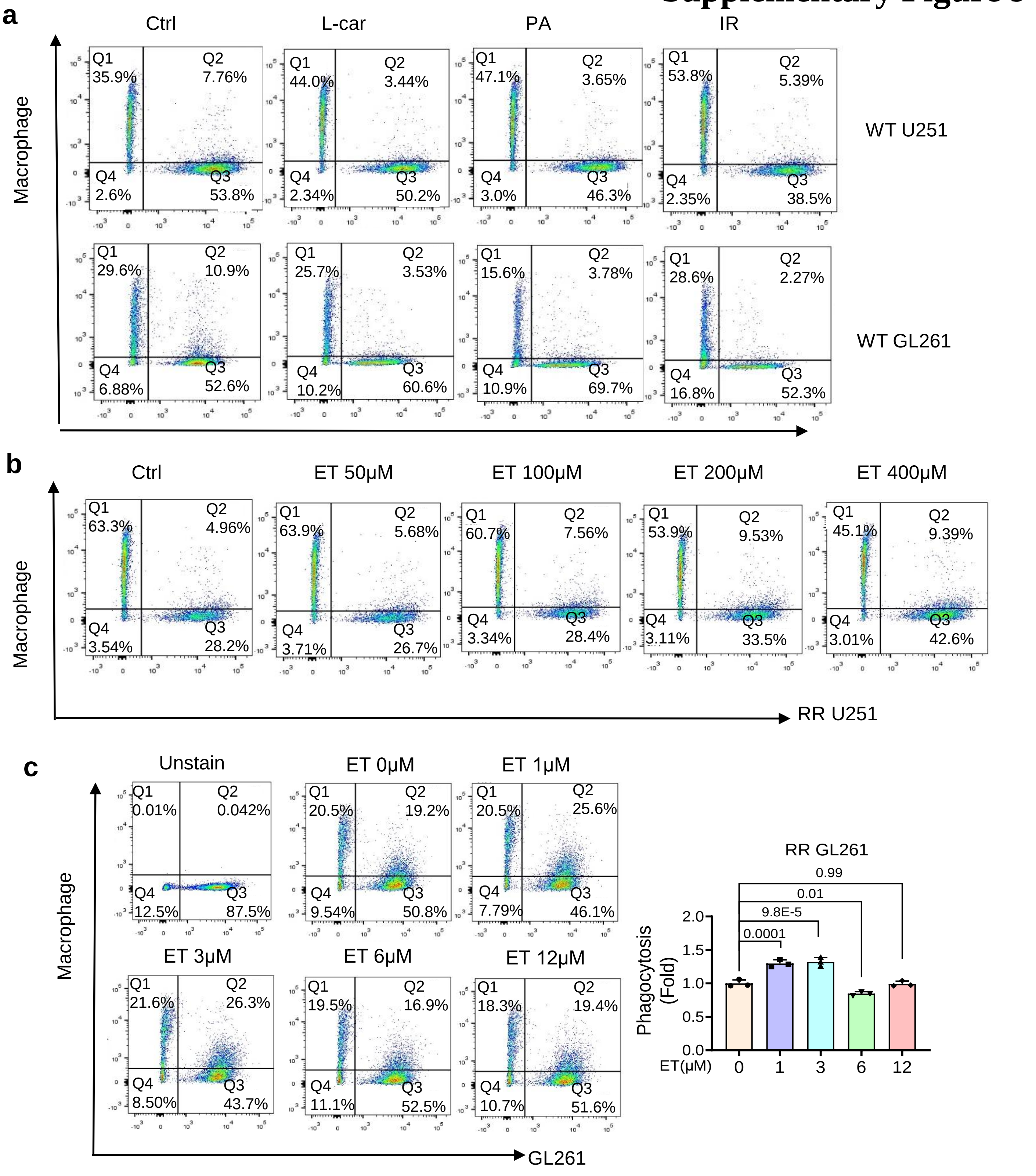

Supplementary Figure 9. Inhibition of FAO sensitizes RR GBM cells. (**a**) Representative flowcytometry of macrophage mediated phagocytosis on WT U251 cells treated with FAO activator L-carnitine (L-car, 10 mM) or lipid enhancer palmitate (PA, 25 μM) for 48 hrs or radiation (IR; 5 Gy, 16 hrs). (**b**) Representative flowcytometry of macrophage mediated phagocytosis on RR U251 cells treated with increasing concentrations of etomoxir (ET). (**c**) Phagocytosis on GL261 cells mediated by mouse phagocytosis treated with indicated concentration of ET(n = 3; Results represent means ± SD ; ANOVA one-way test). Source data are provided as a Source Data file.

Supplementary Figure 10

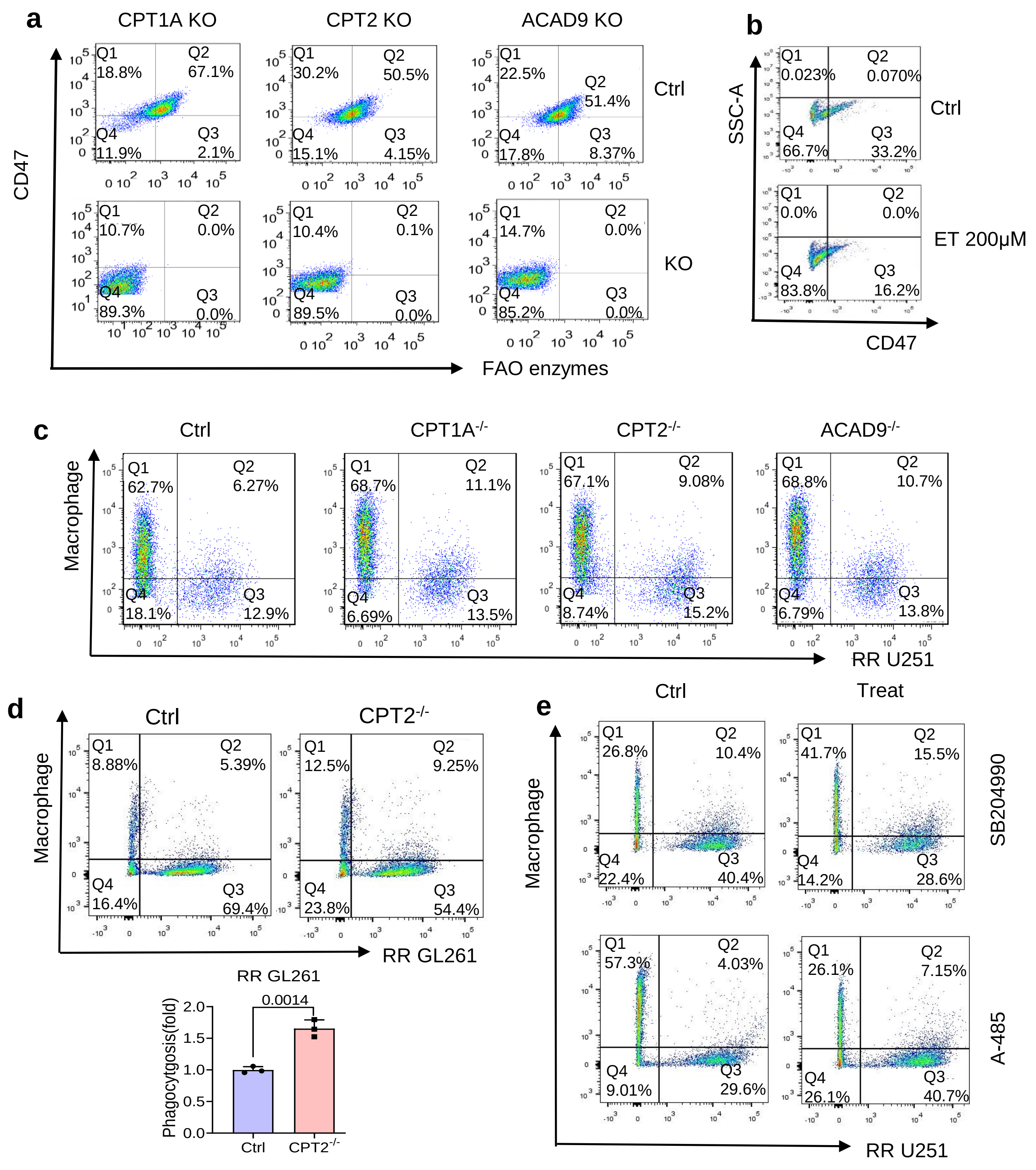

Supplementary Figure 10. (**a**)Representative flowcytometry of CD47 expressing cells in $CPT1A^{-/-}$, $CPT2^{-/-}$, $ACAD9^{-/-}$ RR U251 populations compared to RR U251 cells. (**b**) CD47 positive population detected by flowcytometry in RR U251 cells treated with etomoxir (ET). (**c**) Macrophage(THP1) phagocytosis on vector control and CRISPR-KO $CPT1A^{-/-}$, $CPT2^{-/-}$, $ACAD9^{-/-}$ RR U251 cells detected by flow cytometry. (**d**) Mouse macrophage phagocytosis on vector control and CRISPR-KO CPT2-/- RR GL261 cells detected by flow cytometry (n=3; results represent means ± SD; Two-tailed t test). (**e**) Macrophage(THP1) phagocytosis on RR U251 cells treated with ACLY inhibitor SB 204990 (25 µM, 24 hrs) and A-485 (20 µM, 24 hrs) detected by flow cytometry. Source data are provided as a Source Data file.

Supplementary Figure 11

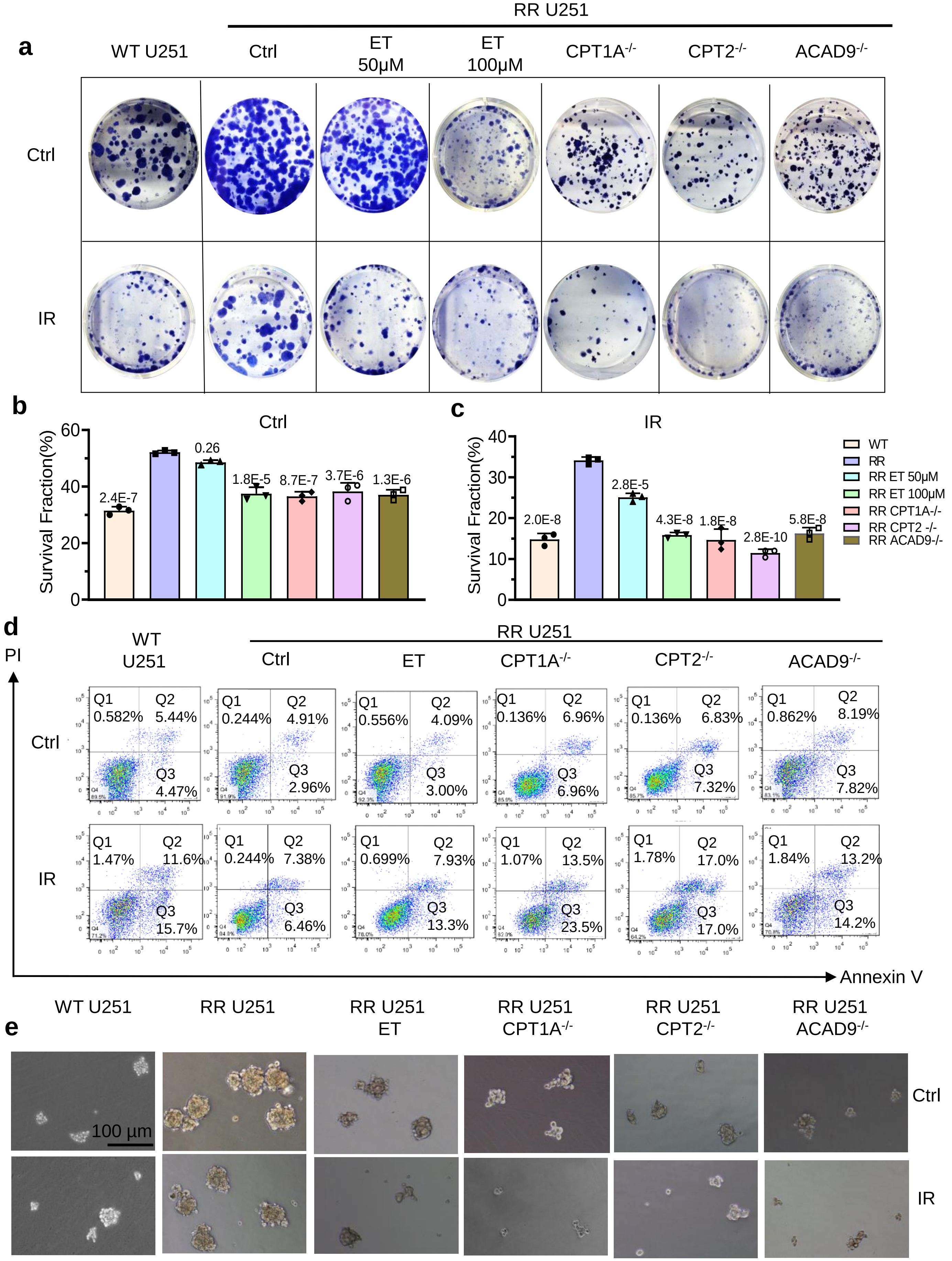

Supplementary Figure 11. Radiation mediated clonogenic suppression and apoptotic cell death were enhanced by ET and in the CRISPR KO CPT1A$^{-/-}$, CPT2$^{-/-}$, ACAD9$^{-/-}$ cells.

(**a**) Representative images of colony formation in WT and RR U251 cells treated with ET (50 or 100 µM 48 h) or CRISPR KO CPT1A$^{-/-}$, CPT2$^{-/-}$, ACAD9$^{-/-}$ cells. (**b, c**) Survival fractions of sham-irradiated (Control, **b**) and irradiated (IR, 5 Gy, **c**) WT U251 cells, CPT1A$^{-/-}$, CPT2$^{-/-}$, ACAD9$^{-/-}$ RR U251 cells and ET (50-100 µM, 48 hrs) treated RR U251 cells compared with RR U251 cells (n = 3 independent experiments; means ± SD; ANOVA one-way test). Representative images of apoptosis (**d**) and neurosphere formation (**e**) of irradiated (5 Gy) WT U251 cells with or without pretreatment with ET (200 µM, 48 hrs) compared to CRISPR KO CPT1A$^{-/-}$, CPT2$^{-/-}$, ACAD9$^{-/-}$ RR U251 cells (scale bar = 100 µm). Source data are provided as a Source Data file.

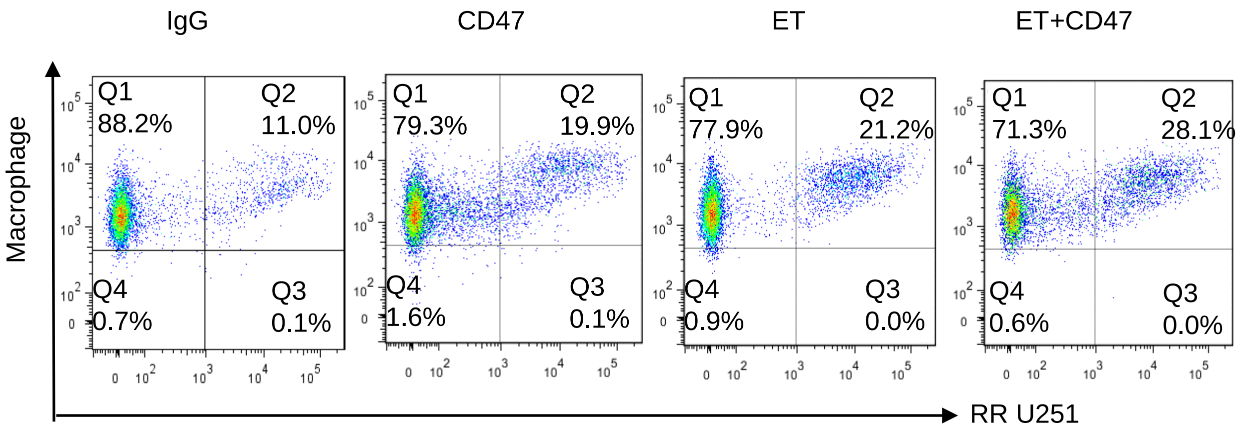

Supplementary Figure 12. In vitro phagocytosis enhanced by anti-CD47 antibody with FAO inhibition. Macrophage phagocytosis was measured with activated THP-1 cells incubated with RR U251 cells treated by ET (400 µM, 12 hrs), anti-CD47 antibody (10 µg/ml, 2 hrs) or combined and normalized with the cells treated with IgG (n = 3).

Supplementary Figure 13

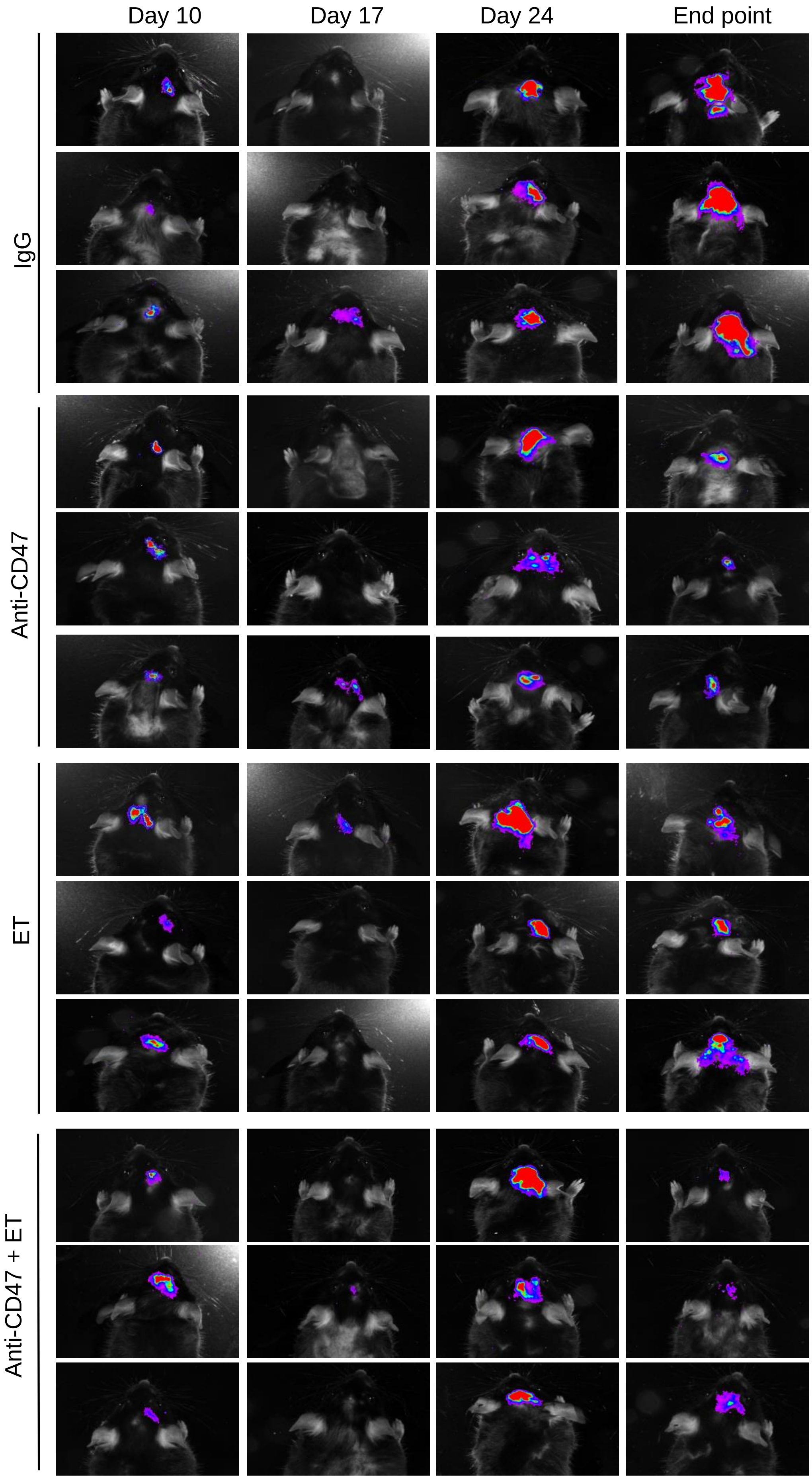

Supplementary Figure 13. Suppression of re-growing orthotopic GBM tumors by radiation with FAO inhibition and anti-CD47 antibody. Additional IVIS images of mouse orthotopic GBM treated with radiation with ET, anti-CD47 antibody or combined.

Supplementary Figure 14

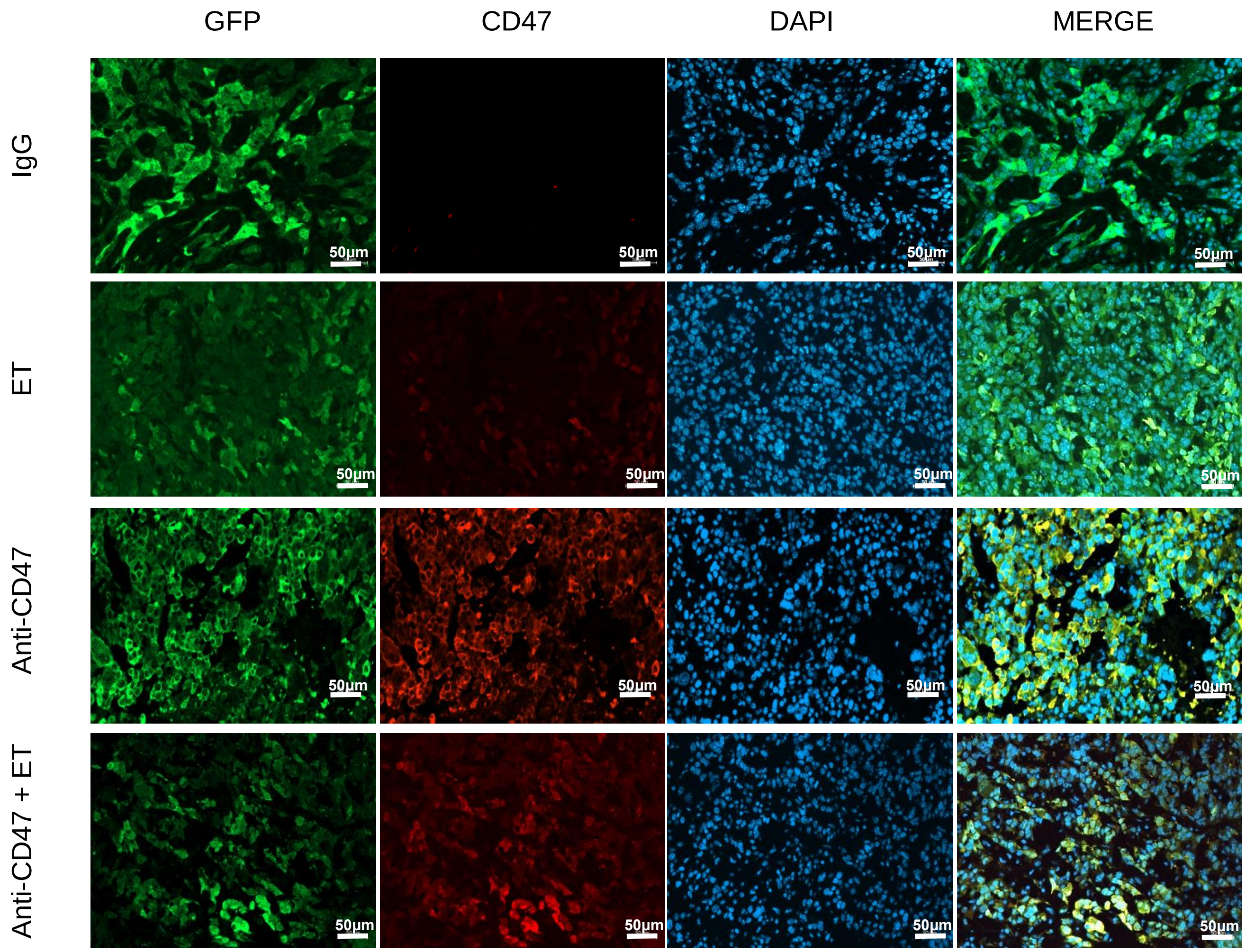

Supplementary Figure 14. Anti-CD47 antibody can penetrate BBB and combine the membrane of GBM cells. Mouse GFP GL261 tumors treated with in vivo radiation (3 Gy x 3) followed by FAO inhibitor ET and/or anti-CD47 antibody with control IgG (n=5; scale bar = 50 µm). IHC immuno-fluorenes staining: Green: Tumor cells with GFP; Red : anti-CD47 antibody attached to the membrane of tumor cells by anti-Rat IgG; Blue: cell nucleus. ET, etomoxir.

Supplementary Figure 15

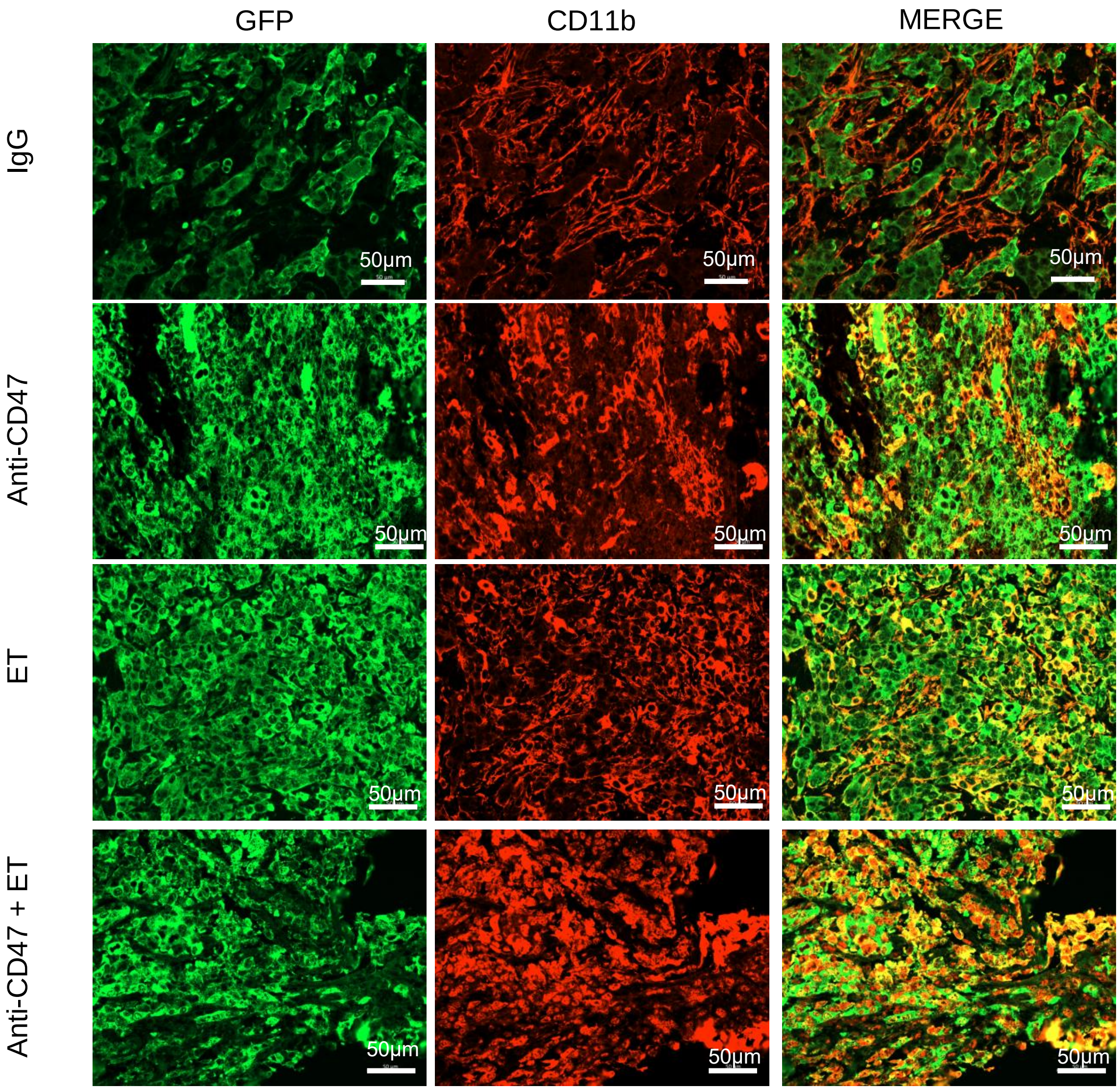

Supplementary Figure 15. Anti-CD47 antibody and FAO inhibition enhanced macrophage phagocytosis on mouse syngeneic GBM. Mouse GFP GL261 tumors treated with in vivo radiation (3 Gy x 3) followed by treatment with control IgG, FAO inhibitor ET and/or anti-CD47 antibody (n=5). IHC immuno-fluorenes staining: Green: tumor cells with GFP; Red: infiltrated macrophages stained by CD11b; ET, etomoxir.

Supplementary Table 1. Expression FAO enzyme (CPT1A, CPT2, ACAD9) and CD47 in 46 HGG patients

Patient NO.	P grade	R grade	Treatment after initial surgery	CD47		CPT1A		CPT2		ACAD9		Time to recurrence (months)
				P	R	P	R	P	R	P	R	
1	Ⅳ	Ⅳ	Radiochemotherapy	1+	2+	1+	3+	1+	3+	1+	1+	9
2	Ⅲ	Ⅲ-Ⅳ	Chemotherapy	1+	2+	1+	3+	0	1+	1+	1+	9
3	Ⅱ-Ⅲ	Ⅱ-Ⅲ	Radiochemotherapy	1+	2+	2+	3+	0	1+	1+	1+	24
4	Ⅱ-Ⅲ	Ⅱ-Ⅲ	Radiochemotherapy	1+	1+	1+	1+	0	1+	1+	2+	11
5	Ⅳ	Ⅳ	Radiochemotherapy	1+	3+	2+	2+	1+	2+	1+	2+	23
6	Ⅳ	Ⅳ	Radiochemotherapy	1+	3+	0	2+	0	1+	0	1+	16
7	Ⅳ	Ⅳ	Radiochemotherapy	1+	3+	1+	3+	1+	2+	1+	2+	13
8	Ⅱ	Ⅳ	N/A	1+	2+	2+	2+	2+	3+	1+	3+	65
9	Ⅳ	Ⅲ	N/A	1+	3+	1+	3+	0	1+	3+	1+	7
10	Ⅲ-Ⅳ	Ⅲ-Ⅳ	Chemotherapy	1+	2+	1+	1+	1+	2+	2+	1+	25
11	Ⅳ	Ⅳ	Radiochemotherapy	1+	3+	2+	2+	2+	1+	1+	2+	9
12	Ⅱ	IV	Gamma knife	1+	2+	2+	3+	0	1+	2+	1+	21
13	Ⅲ	IV	Radiochemotherapy	1+	3+	2+	2+	0	3+	1+	1+	48
14	Ⅲ	Ⅳ	Gamma knife	1+	3+	1+	1+	1+	3+	1+	2+	11
15	Ⅳ	Ⅳ	N/A	1+	2+	1+	3+	1+	3+	1+	1+	3
16	Ⅳ	Ⅳ	Radiochemotherapy	1+	3+	0	1+	0	3+	1+	1+	8
17	Ⅲ	Ⅳ	Chemotherapy	1+	2+	1+	3+	1+	3+	1+	2+	7
18	Ⅳ	Ⅳ	Radiochemotherapy	1+	1+	1+	3+	1+	1+	1+	1+	7
19	Ⅳ	Ⅳ	Radiochemotherapy	1+	2+	1+	3+	2+	3+	1+	2+	16
20	Ⅳ	Ⅳ	Gamma knife	1+	1+	2+	3+	2+	2+	1+	2+	19
21	Ⅳ	Ⅳ	Chemotherapy	1+	3+	1+	3+	1+	3+	1+	2+	5
22	Ⅳ	Ⅳ	Radiochemotherapy	1+	3+	0	3+	0	3+	0	1+	2
23	Ⅳ	Ⅳ	Radiochemotherapy	1+	3+	0	3+	0	3+	1+	2+	7
24	Ⅳ	Ⅳ	Radiochemotherapy	1+	2+	1+	3+	1+	3+	1+	2+	20
25	Ⅳ	Ⅳ	Radiochemotherapy	1+	1+	0	1+	0	1+	1+	3+	6
26	Ⅳ	Ⅳ	Radiochemotherapy	1+	2+	1+	3+	0	2+	1+	2+	17

Patient NO.	P grade	R grade	Treatment after initial surgery	CD47		CPT1A		CPT2		ACAD9		Time to recurrence (months)
				P	R		R	P	R	P	R	
27	Ⅳ	Ⅳ	Chemotherapy	0	2+	0	3+	0	2+	0	3+	5
28	Ⅳ	Ⅳ	Radiochemotherapy	1+	3+	1+	2+	1+	3+	1+	2+	26
29	Ⅳ	Ⅳ	Chemotherapy	1+	1+	2+	3+	0	2+	1+	2+	13
30	Ⅳ	Ⅳ	Radiochemotherapy	1+	3+	2+	0	0	1+	1+	2+	29
31	Ⅱ-Ⅲ	Ⅳ	N/A	1+	2+	1+	3+	0	1+	1+	2+	28
32	Ⅲ-Ⅳ	Ⅲ-Ⅳ	Chemotherapy	1+	2+	0	2+	0	1+	0	1+	23
33	Ⅳ	Ⅳ	Radiochemotherapy	2+	2+	2+	3+	1+	2+	1+	2+	6
34	Ⅳ	Ⅳ	Radiochemotherapy	1+	3+	2+	3+	1+	3+	2+	3+	12
35	Ⅳ	Ⅳ	Radiochemotherapy	0	1+	0	2+	2+	2+	0	2+	1
36	Ⅳ	Ⅳ	Radiochemotherapy	1+	2+	1+	2+	1+	1+	2+	2+	38
37	Ⅳ	Ⅳ	Radiochemotherapy	1+	2+	1+	3+	0	1+	1+	2+	5
38	Ⅳ	Ⅳ	Radiochemotherapy	1+	2+	0	2+	0	3+	1+	1+	5
39	Ⅳ	Ⅳ	Radiochemotherapy	0	2+	1+	3+	0	3+	0	2+	12
40	Ⅳ	Ⅳ	Radiochemotherapy	0	1+	1+	3+	1+	3+	1+	2+	11
41	Ⅱ-Ⅲ	Ⅳ	N/A	1+	1+	1+	2+	0	3+	1+	1+	9
42	Ⅲ	Ⅳ	Radiochemotherapy	1+	1+	1+	3+	0	2+	1+	1+	2
43	Ⅳ	Ⅳ	Radiochemotherapy	1+	1+	1+	3+	1+	2+	1+	2+	11
44	Ⅳ	Ⅳ	Radiochemotherapy	1+	2+	1+	2+	1+	1+	1+	2+	5
45	Ⅳ	Ⅳ	Radiochemotherapy	1+	2+	1+	2+	2+	2+	1+	1+	15
46	Ⅳ	Ⅳ	N/A	1+	2+	2+	0	0	0	1+	2+	2

P, primary; R, recurrent.

Supplementary Table 2. FAO enzymes and CD47 expression in 46 HGG patients with matched primary and recurrent samples

Variable	CD47/ Primary			CD47/ Recurrent		
	Low	High	P-value	Low	High	P-value
Gender (female/male)						
Female	10	9	1	11	8	0.5499
Male	13	14		12	15	
Age	44.39±14.90	43.52±0.76	0.8532	42.09±17.43	45.82±13.03	0.4247
Tumor Size	4.00±0.76	4.5±1.12	0.368	3±0.05	4.72±0.38	0.7747
Location						
frontal	7	13	0.1561	9	11	0.8115
temporal	9	3		7	5	
Parietal	6	5		6	5	
others	1	2		1	2	
CPT1A Status						
Low	13	10	0.3763	11	11	1
High	10	13		12	12	
CPT2 Status						
Low	16	8	0.0182	15	8	0.039
High	7	15		8	15	
ACAD9 Status						
Low	16	7	0.0113	14	9	0.1404
High	7	15		9	14	
PFS(months)	14.52±10.47	14.43±13.09	0.9905			

PFS, Progression-free survival; HGG, high grade glioma. Two-tailed t test was applied in age, tumor size and PFS analysis; Chi-square test was applied in others.

Supplementary Table 3. Cox regression analyses of CD47 and FAO enzyme gene expression related to clinicopathologic characteristics and clinical outcomes

	Univariable regression			Multivariable regression		
	HR	95%CI	P value	HR	95%CI	P value
Gender (female/male)	1.076	0.848-1.365	0.546			
Age(≥50/<50)	1.544	1.214-1.965	<0.001	1.127173	0.853-1.490	0.40029767
Radiotherapy (Yes/No)	0.849	0.595-1.212	0.367			
Chemotherapy (Yes/No)	0.892	0.639-1.245	0.501			
IDH mutation (Yes/No)	0.326	0.252-0.422	<0.001	0.427378	0.315-0.579	4.0489E-08
1p19q codeletion (Yes/No)	0.213	0.133	<0.001	0.383907	0.221-0.666	0.0006553
CD47 (High/Low)	1.745	1.374-2.216	<0.001	1.248242	0.922-1.689	0.15083054
CPT1A (High/Low)	1.295	1.022-10641	0.032	1.015651	0.760-1.358	0.91654933
CPT2 (High/Low)	1.857	1.461-20361	<0.001	1.434148	1.038-1.981	0.02880713
ACAD9 (High/Low)	1.114	0.879-1.411	0.372			
ACADM (High/Low)	1.397	1.101-1.772	0.006	0.707	0.501-0.999	0.049
ACADSB (High/Low)	1.034	0.816-1.310	0.781			

Univariable regression and Multivariable regression analysis were applied.
