# Supplementary material for: Fatty acid oxidation fuels glioblastoma radioresistance with CD47-mediated immune evasion
Source: Nat Commun. 2022 Mar 21;13:1511. doi: 10.1038/s41467-022-29137-3 (PMC8938495; doi:10.1038/s41467-022-29137-3)
Supplement: Supplementary file 3 — Reporting Summary [file 41467_2022_29137_MOESM3_ESM.pdf]

nature research

Corresponding author(s): Jian Jian Li

Last updated by author(s): Jan 26, 2022

Reporting Summary

Nature Research wishes to improve the reproducibility of the work that we publish. This form provides structure for consistency and transparency in reporting. For further information on Nature Research policies, see our Editorial Policies and the Editorial Policy Checklist.

Statistics

For all statistical analyses, confirm that the following items are present in the figure legend, table legend, main text, or Methods section.

n/a	Confirmed	
[ ]	[x]	The exact sample size (*n*) for each experimental group/condition, given as a discrete number and unit of measurement
[ ]	[x]	A statement on whether measurements were taken from distinct samples or whether the same sample was measured repeatedly
[ ]	[x]	The statistical test(s) used AND whether they are one- or two-sided<br>*Only common tests should be described solely by name; describe more complex techniques in the Methods section.*
[x]	[ ]	A description of all covariates tested
[ ]	[x]	A description of any assumptions or corrections, such as tests of normality and adjustment for multiple comparisons
[ ]	[x]	A full description of the statistical parameters including central tendency (e.g. means) or other basic estimates (e.g. regression coefficient) AND variation (e.g. standard deviation) or associated estimates of uncertainty (e.g. confidence intervals)
[ ]	[x]	For null hypothesis testing, the test statistic (e.g. *F*, *t*, *r*) with confidence intervals, effect sizes, degrees of freedom and *P* value noted<br>*Give P values as exact values whenever suitable.*
[x]	[ ]	For Bayesian analysis, information on the choice of priors and Markov chain Monte Carlo settings
[x]	[ ]	For hierarchical and complex designs, identification of the appropriate level for tests and full reporting of outcomes
[ ]	[x]	Estimates of effect sizes (e.g. Cohen's *d*, Pearson's *r*), indicating how they were calculated

Our web collection on statistics for biologists contains articles on many of the points above.

Software and code

Policy information about availability of computer code

Data collection	1. The matrix and raw data for RNA-seq reported in this paper have been deposited in Chinese Glioma Genome Atlas organization (CGGA,http://www.cgga.org.cn/) and are accessible through dataset ID mRNAseq_693.<br>2. The sgRNAs were designed using the CRISPR design software (http://crispr.mit.edu) following the instructions published by Dr. Zhang's Lab;
Data analysis	1. The RNA-seq data was normalized for analyzing relative expression.<br>2.The overall survival (OS) of GBM patients treated by radiotherapy and other methods was evaluated using the Kaplan–Meier method.<br>3. The statistical differences in survival length were determined using the log-rank test.<br>4. Data generated from RNAseq and the clustering and correlation analyses were performed using the ggplots, fgesa and limma R language packages, respectively.<br>5. FlowJo v10.0.7 (BD) was used for flow cytometry data analysis.<br>6. Image Pro Plus 6.0 was used for quantitation of macrophage-mediated phagocytosis.<br>7. Graphpad, Microsoft Excel and SPSS were used for drawing pictures, analyzing data, and statistics.<br>8. Microsoft Power Point was used for organizing the data presentation.

For manuscripts utilizing custom algorithms or software that are central to the research but not yet described in published literature, software must be made available to editors and reviewers. We strongly encourage code deposition in a community repository (e.g. GitHub). See the Nature Research guidelines for submitting code & software for further information.

Data

Policy information about availability of data

All manuscripts must include a data availability statement. This statement should provide the following information, where applicable:

- Accession codes, unique identifiers, or web links for publicly available datasets
- A list of figures that have associated raw data
- A description of any restrictions on data availability

1. Figures 1-8 are with raw data. There are no restrictions on data availability.
2. Extended data are presented as Supplementary Figures 1-15 and Supplementary Tables 1-3. There are no restrictions on data availability
3. The paired human glioma tissues samples were obtained from Xiangya Hospital, Central South University in China. All the 46 patients underwent intracranial surgery from March 2010 to January 2019; and 34 out of these patients received radiotherapy after first surgery.
4. The matrix and raw data for RNAseq reported in this paper have been deposited in Chinese Glioma Genome Atlas organization (CGGA,http://www.cgga.org.cn/) and are accessible through dataset ID mRNAseq_693. All other data are available from the corresponding author upon reasonable request.

Field-specific reporting

Please select the one below that is the best fit for your research. If you are not sure, read the appropriate sections before making your selection.

[x] Life sciences [ ] Behavioural & social sciences [ ] Ecological, evolutionary & environmental sciences

For a reference copy of the document with all sections, see nature.com/documents/nr-reporting-summary-flat.pdf

Life sciences study design

All studies must disclose on these points even when the disclosure is negative.

Sample size	1. The sample sizes of in vitro experiments were determined based on our previous studies.<br>2. A total 46 paired GBM tumors (total tumor number n = 92) was applied in evaluating FAO enzymes and CD47 expression associated with clinical outcomes (Supplementary Tables 1-3).<br>3. For in vivo mouse experiments, 5-7 mice is included in each testing group and the control group.
Data exclusions	We have no other data exclusions in this study.
Replication	1. For western blots, each experiment was repeated at least twice independently.<br>2. For flow cytometry analysis, each experiment was performed 3 times independently.<br>3. Experiments using immunocytochemistry, luciferase reporters, cell phagocytosis, clonogenic survival, gap filling rate, neurosphere formation and trans-well invasion assays were all repeated for at least 3 times independently.<br>4. Datas from CGGA data base (RNA-seq from 504 gliomas) were consistent with the observation from our own data.
Randomization	1. Mice that were inoculated with tumor cells were randomly divided into groups before subsequent treatments.<br>2. The data analysis of IHC, Neurosphere is based on at least 6 randomly taken images form each sample.
Blinding	All GBM-unrelated informations and treatment history for the GBM patients were blocked in the clinic studies. Investigators were not blinded during tumor inoculation and animal grouping. For IHC staining of tumor tissues, the investigators at the pathology core service were blinded for the clinical information of each sample.

Reporting for specific materials, systems and methods

We require information from authors about some types of materials, experimental systems and methods used in many studies. Here, indicate whether each material, system or method listed is relevant to your study. If you are not sure if a list item applies to your research, read the appropriate section before selecting a response.

Materials & experimental systems

n/a	Involved in the study	
[ ]	[x]	Antibodies
[ ]	[x]	Eukaryotic cell lines
[x]	[ ]	Palaeontology and archaeology
[ ]	[x]	Animals and other organisms
[x]	[ ]	Human research participants
[x]	[ ]	Clinical data
[x]	[ ]	Dual use research of concern

Methods

n/a	Involved in the study	
[x]	[ ]	ChIP-seq
[ ]	[x]	Flow cytometry
[ ]	[x]	MRI-based neuroimaging

Antibodies

Antibodies used

CD133 MiltenyiBiotec□ 130-080-801□ FCM (1:50)
CD133 Boster(PA2049) WB (1:1000)
HER2 R&D systems (FAB1129A) FCM (1:50)
HER2 Santa Cruz (sz284) WB (1:1000)
OCT4 Cell Signaling Technology□ 2840□ WB (1:500)
SOX2 Cell Signaling Technology□ 3579□ WB (1:500)
NANOG Cell Signaling Technology□ 4903□ WB (1:500)
β-actin Sigma□ A2066□ WB (1:12000)
CPT1A Cell Signaling Technology□ 12252□ WB (1:500), IF(1:50), IHC (1:50)
CPT2 Santa Cruz (SC-377294) WB (1:500), IF (1:100), IHC (1:80)
anti-rabbit IgG Cell Signaling Technology□ 7074s□ WB (1:2000)
anti-mouse IgG Cell Signaling Technology□ 7076s□ WB (1:3000)
CD47 BD pharmingen (556045) FCM (1:50)
CD47 Boster (PA2223) WB (1:1000)
CD47 Santa Cruz (sc-12730) IHC (1:80)
CD47 Hu5F9-G4 Forty-Seven company (3313-06062018)
CD11b Thermo Fisher (MA5-17857) IF (1:100)
RelA-K310AC abcam (ab19870) WB (1:500)
RelA(p65) Santa Cruz(sc-372) WB (1:1000)
Histone H3ac (K9 + K14 + K18 + K23 + K27) abcam (ab47915) WB (1:500)
Histone H3(D1H2) Cell signaling(4499) WB(1:2000)
Acetylated-Lysine Cell signaling(9441) WB(1:1000)

Validation

All antibodies are from commercial sources and have been validated according to previous publications and related manufacturer's information.

Eukaryotic cell lines

Policy information about cell lines

Cell line source(s)

Human monocyte THP1 cells and GBM U87 cells were purchased from American Type Culture Collection. U251 and GL261 cell lines were provided By Dr. Kit Lam's lab at UC Davis. A172 cells were kindly provided by Dr. Rajesh Khanna at College of Medicine, University of Arizona.

Authentication

U251, GL261 and A172 cells were authenticated by Short Tandem Repeat (STR) analysis.

Mycoplasma contamination

All cell lines were tested negative for mycoplasma contamination.

Commonly misidentified lines
(See ICLAC register)

N/A

Animals and other organisms

Policy information about studies involving animals; ARRIVE guidelines recommended for reporting animal research

Laboratory animals

C57BL/6 mice; nude mice

Wild animals

The study did not use wild animals.

Field-collected samples

The study did not involve samples collected from the filed.

Ethics oversight

Animal use and care protocol of in vivo radiation treatment was approved by the Institutional Animal Use and Care Committee of the University of California Davis (IACUC 15315).

Note that full information on the approval of the study protocol must also be provided in the manuscript.

Flow Cytometry

Plots

Confirm that:

- [x] The axis labels state the marker and fluorochrome used (e.g. CD4-FITC).
- [x] The axis scales are clearly visible. Include numbers along axes only for bottom left plot of group (a 'group' is an analysis of identical markers).
- [x] All plots are contour plots with outliers or pseudocolor plots.
- [x] A numerical value for number of cells or percentage (with statistics) is provided.

Methodology

Sample preparation	The apoptosis assay, phagocytosis assay and FACS were descriptived in "Method" section.
Instrument	The FACS Canto II cytometer (BD)
Software	FlowJo v10.0.7 (BD)
Cell population abundance	This project is not involved with Cell sorting procedure.
Gating strategy	To identify the cells population of interesting and exclude the dead cells and cells debris, the forward vs side scatter (FSC-A versus SSC-A) was used. The FSC-A/FSC-H were used to remove the doublets from total cell population. Positive cells were gated out based on gating of unstained cells and single stained cells using appropriate channels.

- [x] Tick this box to confirm that a figure exemplifying the gating strategy is provided in the Supplementary Information.

Magnetic resonance imaging

Experimental design

Design type	tumor imaging in syngeneic mouse orthotopic GBM model.
Design specifications	T1 weight enhancement scans were acquired to detect tumor sizes were monitored at day 10, day 17 and day 28.
Behavioral performance measures	N/A

Acquisition

Imaging type(s)	Structural MRI
Field strength	7.0 Tesla
Sequence & imaging parameters	For in vivo imaging , coronal slice were acquired in a single imaging sesion. T1 weight images were obtained using a fast spin echo pulse sequence with excitation and refocusing flip angles of 90 and 180° respectively. In each scan(TR/TE = 300/14 ms), 7 slices per brain volume were scanned in a field of view (FOV) 8.0 x 8.0 cm2, with a matrix size of 256x 256 pixels with a slice thickness of 1.0 mm and an interslice distance of 1.5 mm, taking two averages per slice with a scan time per brain volume of 1 min 16 s.
Area of acquisition	Multiple slices were taken that covered the whole brain

Diffusion MRI ☐ Used ☒ Not used

Preprocessing

Preprocessing software	Paravision 4
Normalization	Not applicable
Normalization template	Not applicable
Noise and artifact removal	Not applicable
Volume censoring	The volume was measured by software Paravision 4

Statistical modeling & inference

Model type and settings	We performed univariate analysis on the relative tumor area. Relative tumor area was calculated as the product of the

size of the aberrant growth along the dorsal-ventral and medial-lateral axises. This analysis was performed at a fixed location along the rostral-caudal axis. Analysis was done across fixed time points: day 10, day 17 and day 28.

Effect(s) tested

No behavior effects were tested. The only differences across cohorts were therapeutic differences. All the animals were treated the same prior to, during, and after the MRI recording session.

Specify type of analysis: ☐ Whole brain ☒ ROI-based ☐ Both

Anatomical location(s)

Regions of interest (ROIs) drawn by the same experienced radiologist manually on the mentioned regions were identified and the average signal intensity values were calculated automatically from the Paravision 4 software. The ROI of subcutaneous tumour had an area of 20~30mm2. For orthotopic brain tumour, the ROI was 1~1.5 mm2. The ROI was selected by avoiding tumour blood vessel, tumour necrosis and cerebrospinal fluid.

Statistic type for inference
(See Eklund et al. 2016)

N/A

Correction

No correction methods were used

Models & analysis

n/a	Involved in the study
☒	☐ Functional and/or effective connectivity
☒	☐ Graph analysis
☒	☐ Multivariate modeling or predictive analysis
